# Supplementary material for: Thermal Post-Cross-Linking of Siloxane/Silsesquioxane Hybrids with Polycyclic Aromatic Units for Tailored Softening Behavior in High-Temperature Applications
Source: Molecules. 2025 Aug 29;30(17):3532. doi: 10.3390/molecules30173532 (PMC12430222; doi:10.3390/molecules30173532)
Supplement: Supplementary file 1 [file molecules-30-03532-s001.zip › molecules-3809201-supplementary.docx]

**Supporting Information**

**Thermal Post-Cross-Linking of Siloxane/Silsesquioxane Hybrids with Polycyclic Aromatic Units for Tailored Softening Behavior in High-Temperature Applications** Max Briesenick, and Guido Kickelbick*

Saarland University, Inorganic Solid-State Chemistry, Campus, Building C4 1, 66123 Saarbrücken, Germany

E-Mail: guido.kickelbick@uni-saarland.de

Table of Contents

[1 Synthesis of Dimethoxyphenyl‑(1‑naphthyl)silane 2](#_Toc204323836)

[2 Differential Scanning Calorimetry (DSC) 3](#_Toc204323837)

[3 Thermal Treatment Experiment 4](#_Toc204323838)

[4 Dynamic Mechanical Analysis (DMA) 5](#_Toc204323839)

[5 Nuclear Magnetic Resonance (NMR) Spectroscopy 7](#_Toc204323840)

[6 Fourier Transform Infrared (FTIR) Spectroscopy 19](#_Toc204323841)

[7 Powder X-ray Diffraction (PXRD) 20](#_Toc204323842)

[8 Ultraviolet-visible (UV-vis) Spectroscopy 22](#_Toc204323843)

[9 References 24](#_Toc204323844)

# Synthesis of Dimethoxyphenyl‑(1‑naphthyl)silane

**
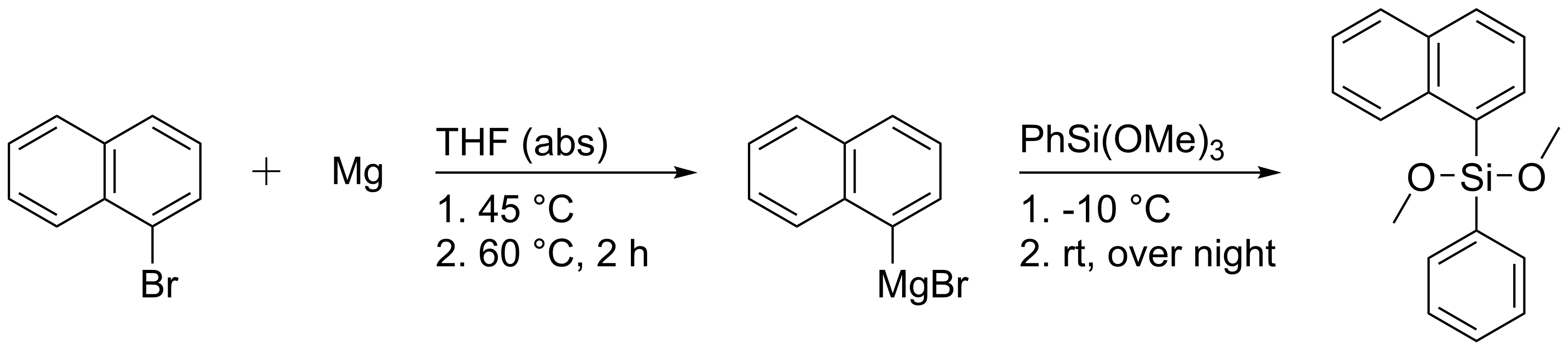
**

**Scheme S 1:** Synthesis of 1‑NaphPhSi(OMe)_2_ using a Grignard reaction.

The synthesis of dimethoxyphenyl‑(1‑naphthyl)silane was done according to our previous publication using a Grignard reaction.^1^

To a 1000 mL three-necked round bottom flask, which was equipped with a reflux condenser, a dropping funnel and a gas inlet and was flame‑dried under vacuum and back‑filled with argon magnesium chips (12.37 g, 508.8 mmol, 1.5 eq) and 400 mL abs THF were added and heated to 45 °C. 1‑bromonaphthalene (70.39 g, 339.9 mmol, 1 eq) was diluted with 60 mL of abs THF in a dropping funnel an added dropwise over 30 minutes. After the addition the reaction mixture was stirred at 60 °C for 2 h. To a 1000 mL Schlenk flask, which was flame‑dried under vacuum and back‑filled with argon phenyltrimethoxysilane (203.4 g, 1025.7 mmol, 3 eq) was added and cooled to ‑10 °C in an ethanol/nitrogen bath. The hot reaction mixture was added to the cooled phenyltrimethoxysilane via cannula in one swoop while stirring, allowed to warm to room temperature and stirred overnight.

After the solvent was removed under vacuum 400 mL n‑hexane were added, refluxed for 10 minutes, allowed to cool to room temperature and decanted. 200 mL n‑hexane were added to the remaining solid, refluxed again for 10 minutes and filtered while hot. The solvent of the combined organic layers was removed under vacuum and the remaining solution was distillated: 1. Fraction (1.3x10^-2^ mbar, 80 °C) was excess phenyltrimethoxysilane, 2. Fraction (1.3x10^‑2^ mbar, 170 °C) was the desired product. The product solidified overnight, was mortared, washed with cold ethanol, and also precipitated from said ethanol. Dimethoxyphenyl‑(1‑naphthyl)silane was obtained with a yield of 74.3 g (74 %) as a white powder.

# Differential Scanning Calorimetry (DSC)





**Figure S1:** DSC measurements of all siloxanes after consolidation. Each sample was measured three times.

# Thermal Treatment Experiment


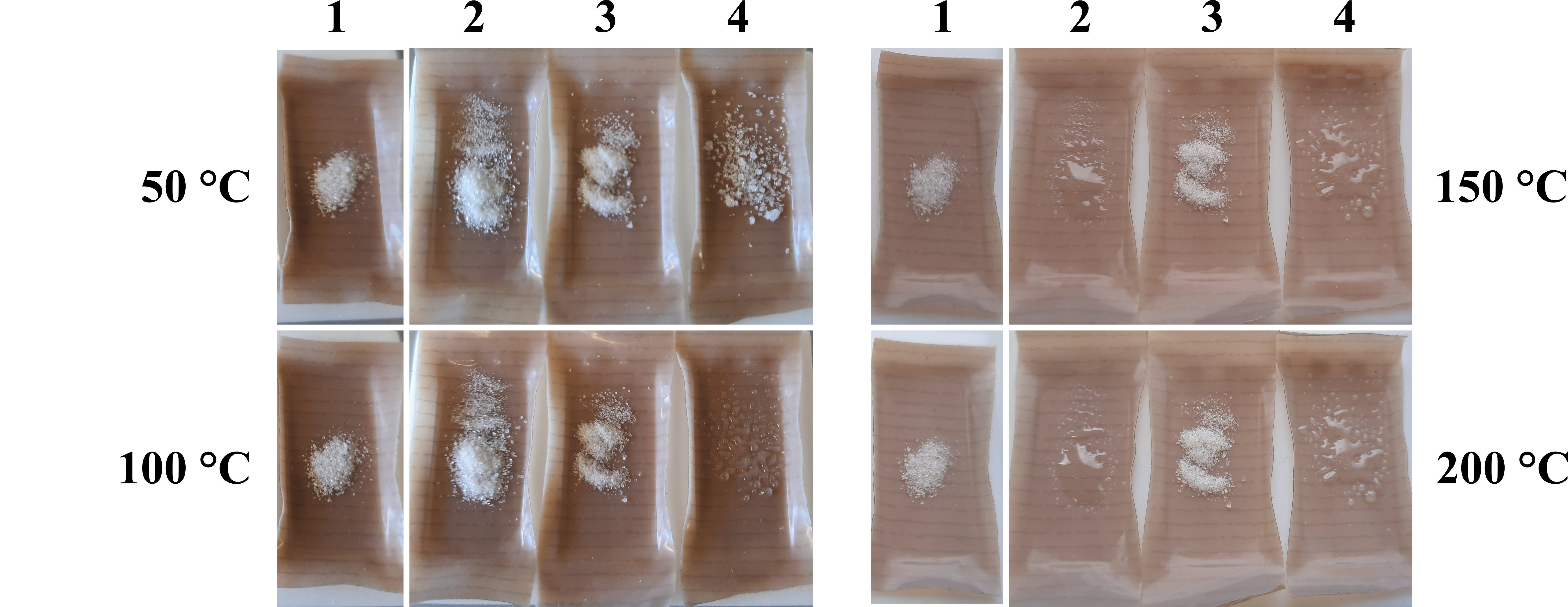


**Figure S2:** Thermal treatment experiments of all solid siloxane samples at different temperatures (only temperatures are shown at which samples turned liquid). 1) NaphMG_2Ph_2_, 2) NaphMG_5Ph_d, 3) NaphMG_4Ph, 4) NaphMG_Sn.

# Dynamic Mechanical Analysis (DMA)





**Figure S3:** Dynamic mechanical analysis of all siloxanes. a) tan δ, b) tan δ (zoomed in), c) storage modulus, d) storage modulus (zoomed in), e) loss modulus, f) loss modulus (zoomed in), g) viscosity, h) viscosity (zoomed in).

**
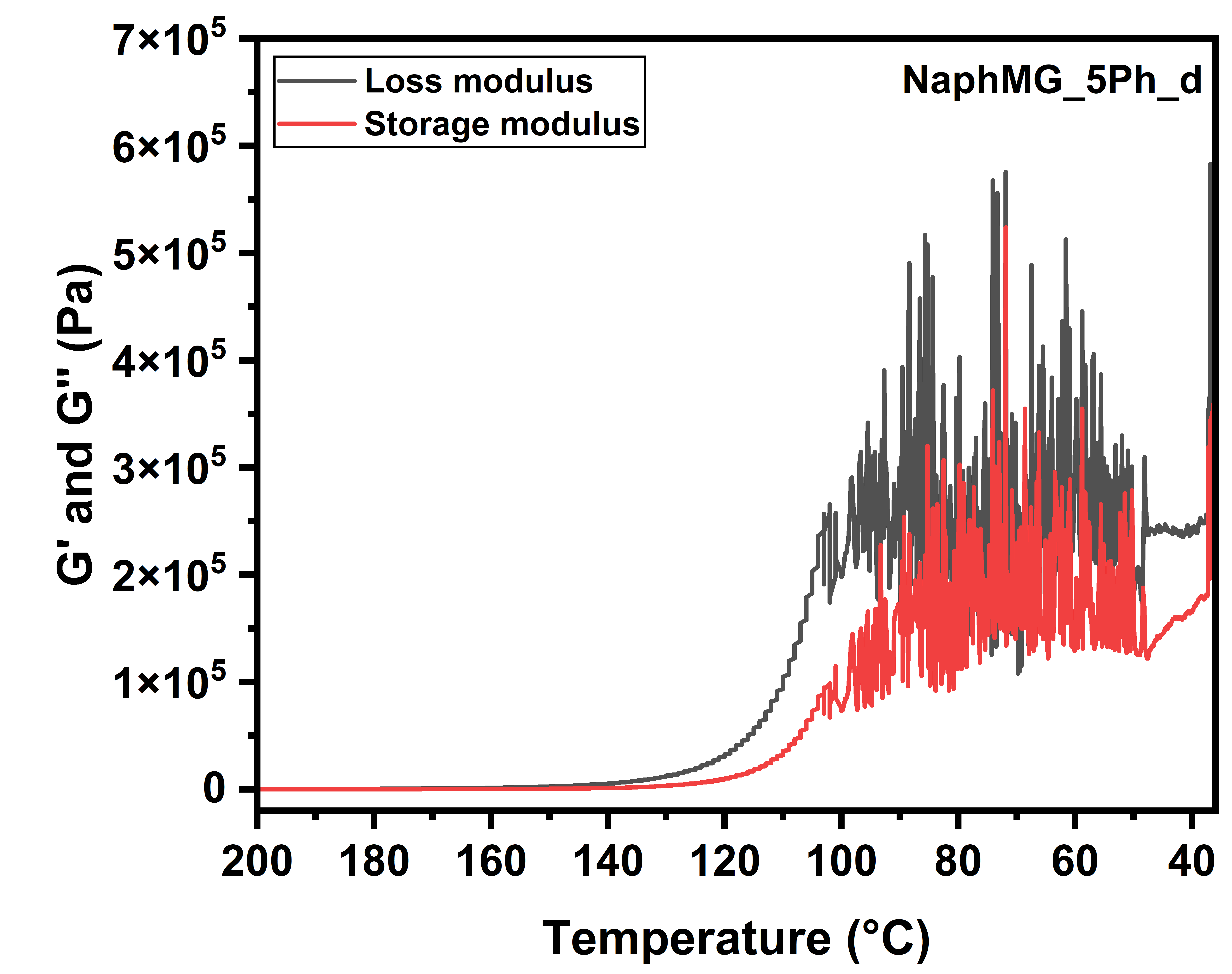
**

**Figure S4:** Storage (G’) and loss modulus (G’’) of NaphMG_5Ph_d across the whole temperature range.

**Table S1:** Viscosities of all siloxanes. a) not measured due to viscosity being too high and therefore risk of breakage, b) not measured due to viscosity being too low and therefore risk of loss of contact between both plates. c) Viscosities are not reliable due to cracking/breaking of the sample.

| **Amount viscosity (mPa∙s)** | | | | | | | |
| --- | --- | --- | --- | --- | --- | --- | --- |
|  | **50  °C** | **100 °C** | **150  °C** | **200  °C** | **150  °C** | **100  °C** | **50  °C** |
| **NaphMG_2Me_2_** | 3350 | 229 | 55.4 | b | b | 233 | 3490 |
| **NaphMG_2Ph_2_** | a | a | a | 479000 | 1400000 | 3430000^c)^ | 260000^c)^ |
| **NaphMG_4Ph** | a | a | a | 1610000 | 1570000 | 2590000 | 2.41E7^c)^ |
| **NaphMG_5Ph_d** | a | a | 395000 | 26800 | 417000 | 3.38E7^c)^ | 4.03E7^c)^ |
| **NaphMG_cond.** | 18200 | 338 | 58.7 | b | b | 416 | 43500 |
| **NaphMG_cons.** | 654000 | 2930 | 322 | b | b | 3190 | 733000 |
| **NaphMG_Sn** | a | a | 510000 | 416000 | 557000 | 1130000 | 4220000 |

# Nuclear Magnetic Resonance (NMR) Spectroscopy

Besides ^29^Si- and ^13^C CP‑MAS NMR spectroscopy we also applied ^29^Si‑ and ^13^C SP‑MAS spectroscopy as a comparison because the former tends to underestimate the amount of highly cross‑linked species.^2–4^ Since these two techniques can only be applied to solid samples, the viscous sample NaphMG_2Me_2_ could not be measured like this. The solid NaphMG could also not be measured using solid state NMR since the pulverized sample did not rotate properly in the NMR due to being sticky at room temperature and a stationary measurement showed only very broad and overlapping signals that could not be analyzed properly. Therefore, ^29^Si solution NMR measurements were conducted to these two samples using chromium(III)acetylacetonate as a relaxation agent.^5–7^ Unfortunately, NaphMG_Sn did show the same problems in the solid state NMR and also did not sufficiently dissolve in any NMR solvent, leading to no measurement available.

The DOC of all samples was calculated according to equation (1’).^8^

|  | $DOC=\frac{D^{1}+2D^{2}+D^{1´}+{2D}^{2´}+T^{1}+{2T}^{2}+{3T}^{3}}{2\cdot\left( D^{1}+D^{2}+D^{1´}+D^{2´} \right)+3\cdot\left( T^{1}+T^{2}+T^{3} \right)}$ | (1’) |
| --- | --- | --- |

**NaphMG**


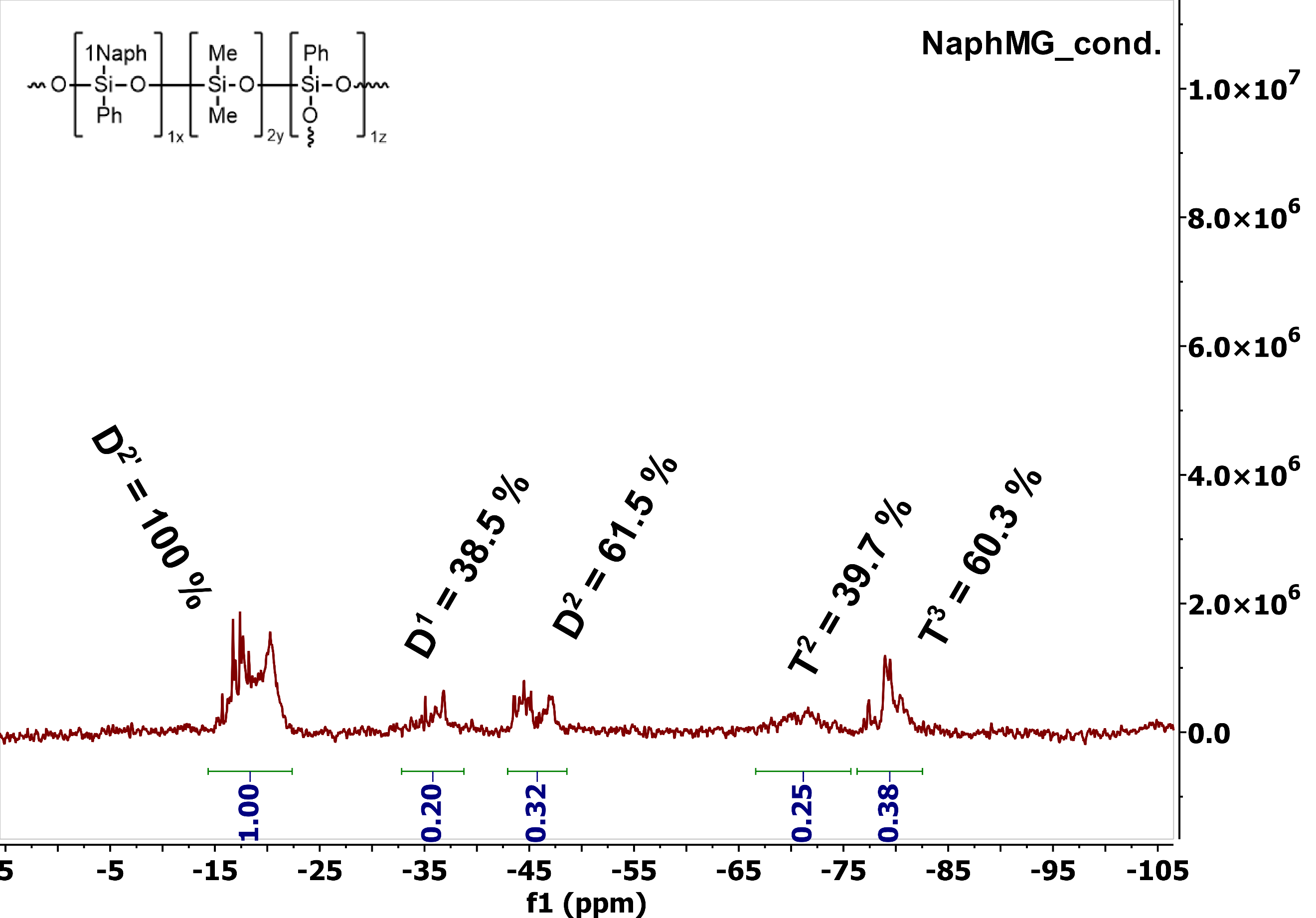


**Figure S5:** ^29^Si NMR spectrum (CDCl_3_, 59.63 MHz, liquid) of NaphMG before consolidation.


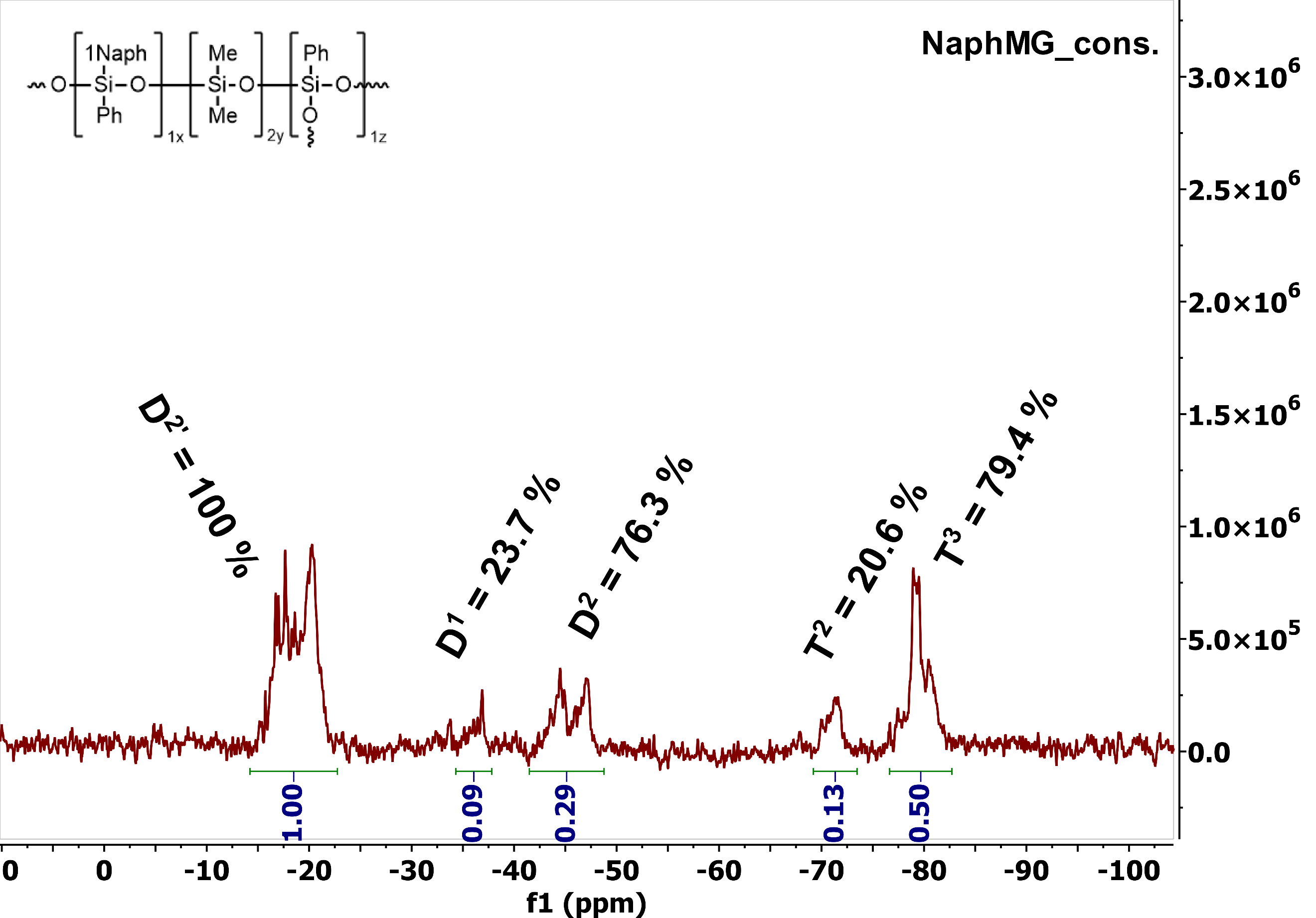


**Figure S6:** ^29^Si NMR spectrum (CDCl_3_, 59.63 MHz, liquid) of NaphMG after consolidation.

**NaphMG_2Me_2_**


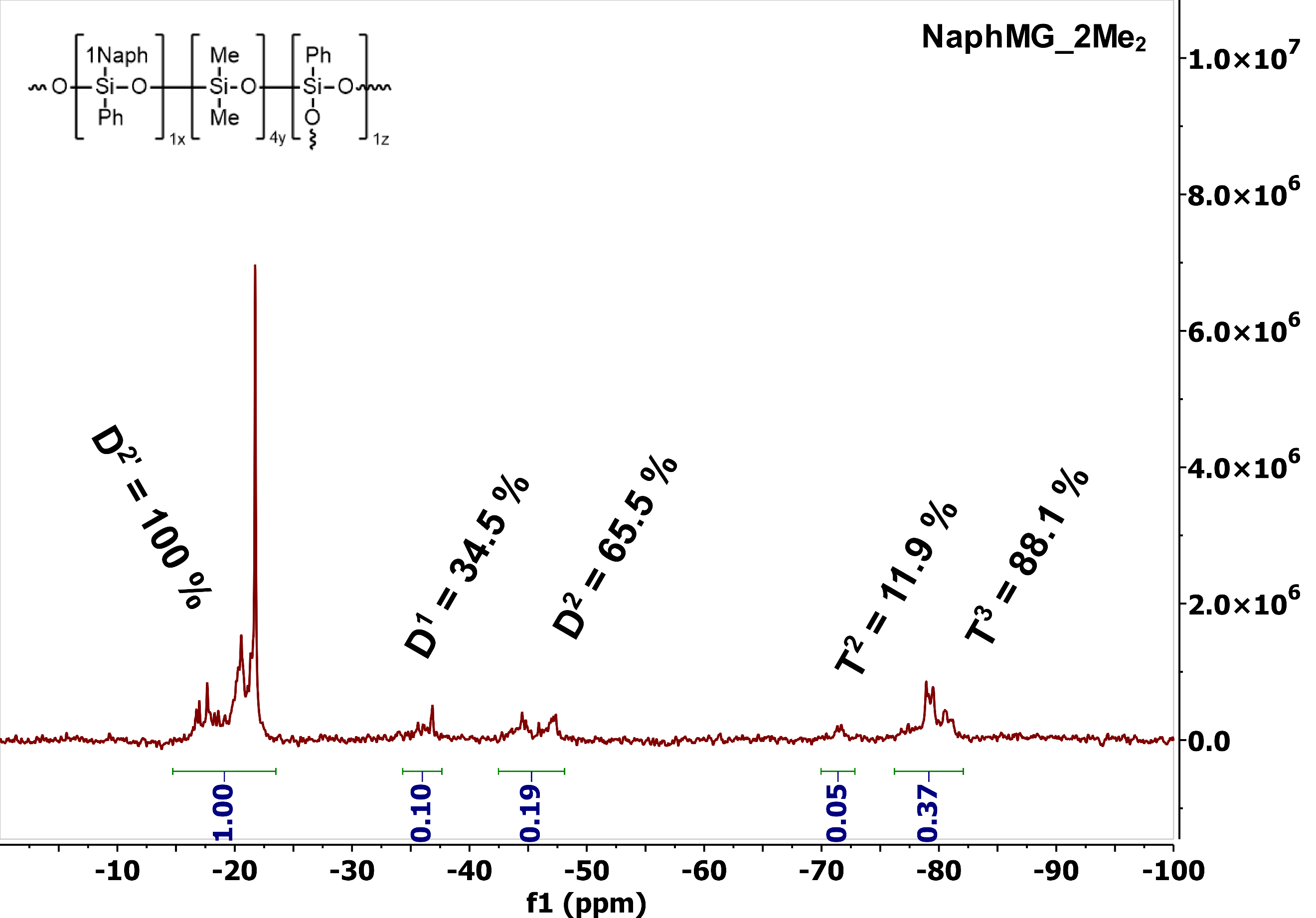


**Figure S7:** ^29^Si NMR spectrum (CDCl_3_, 59.63 MHz, liquid) of NaphMG_2Me_2_ after consolidation.

**NaphMG_2Ph_2_**


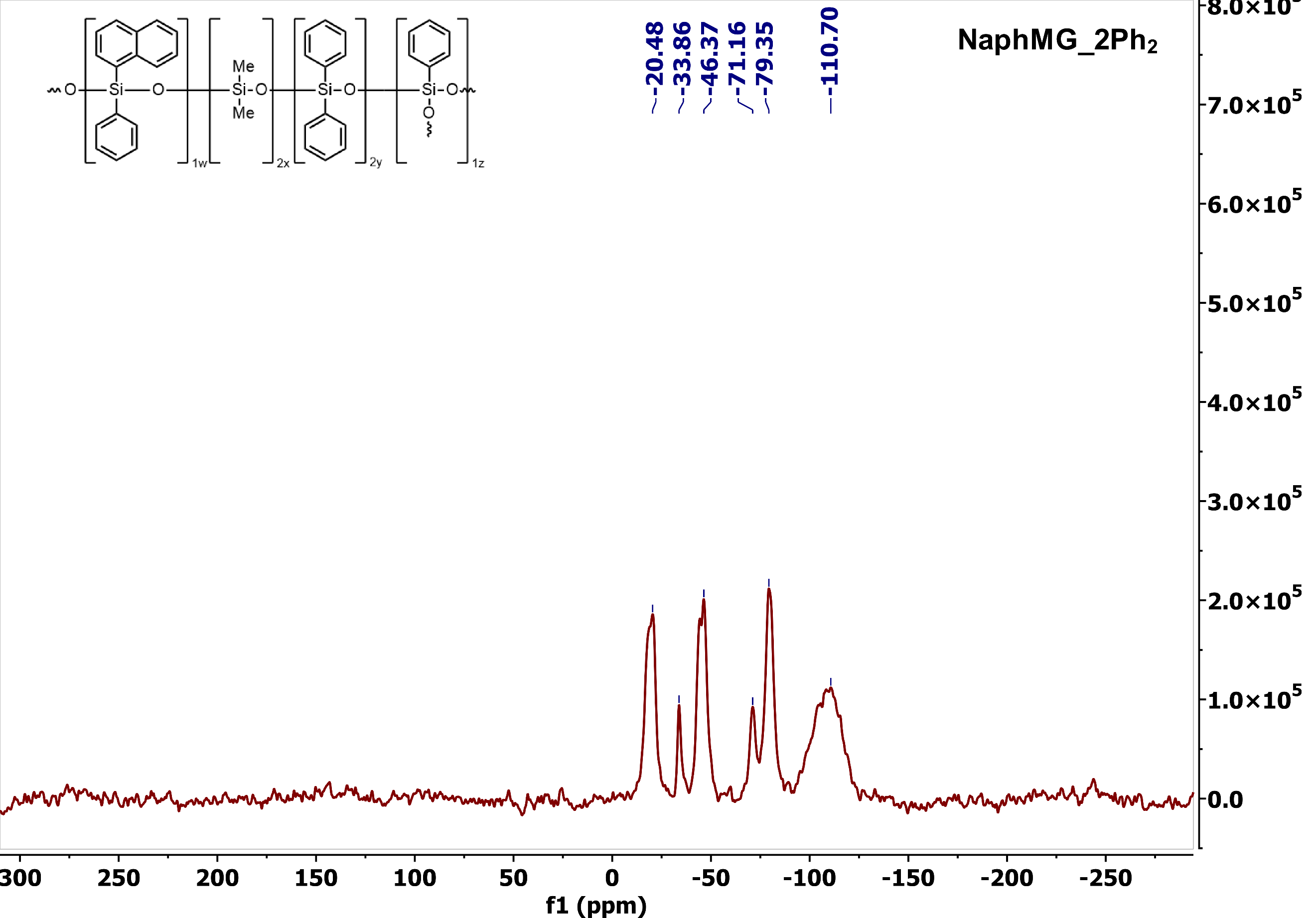


**Figure S8:** ^29^Si MAS spectrum of NaphMG_2Ph_2_ after consolidation.


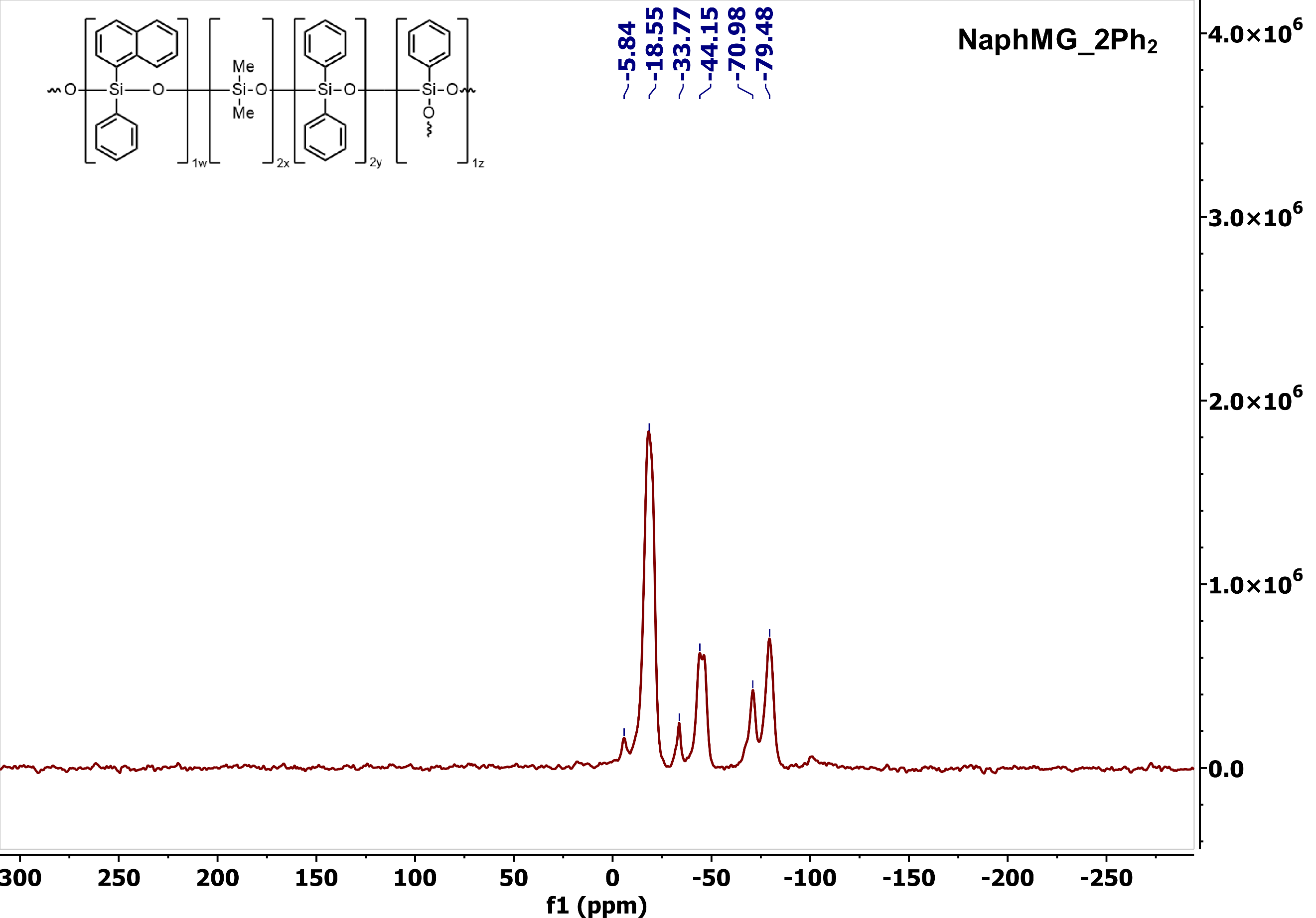


**Figure S9:** ^29^Si CP-MAS spectrum of NaphMG_2Ph_2_ after consolidation.





**Figure S10:** Integrated ^29^Si MAS spectrum of NaphMG_2Ph_2_ after consolidation.





**Figure S11:** Integrated ^29^Si CP-MAS spectrum of NaphMG_2Ph_2_ after consolidation.


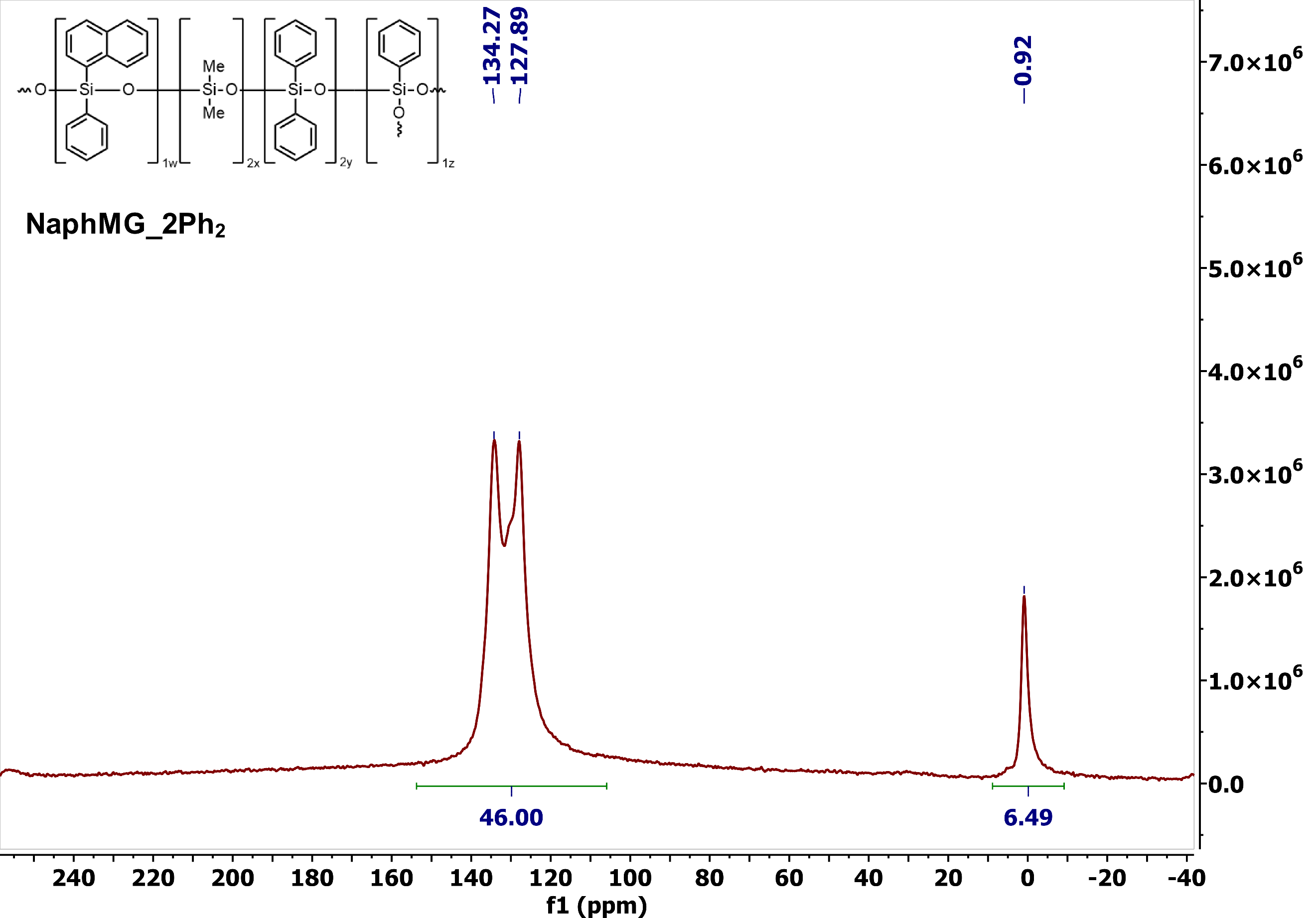


**Figure S12:** ^13^C MAS spectrum of NaphMG_2Ph_2_ after consolidation.


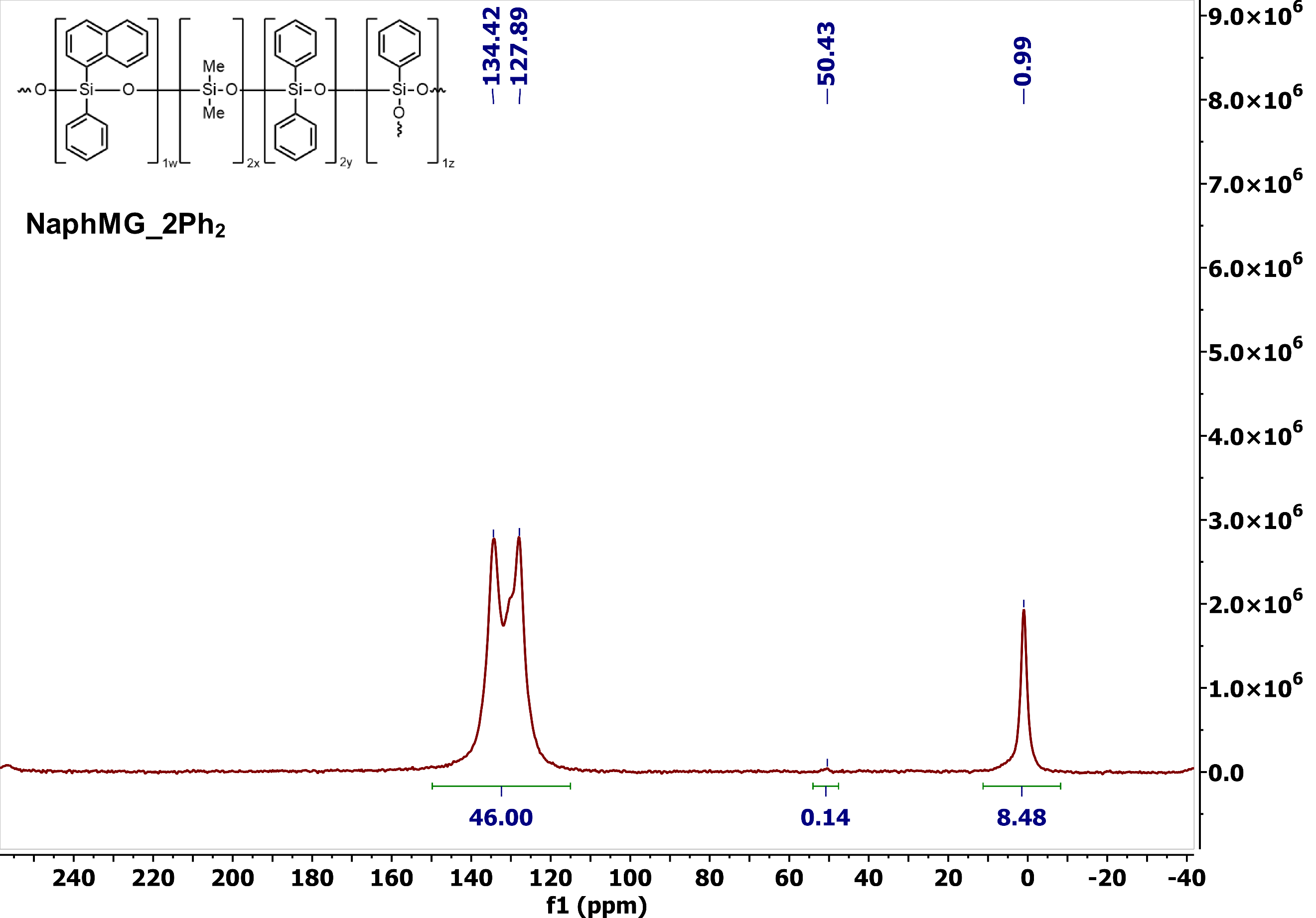


**Figure S13:** ^13^C CP-MAS spectrum of NaphMG_2Ph_2_ after consolidation.

**NaphMG_4Ph**


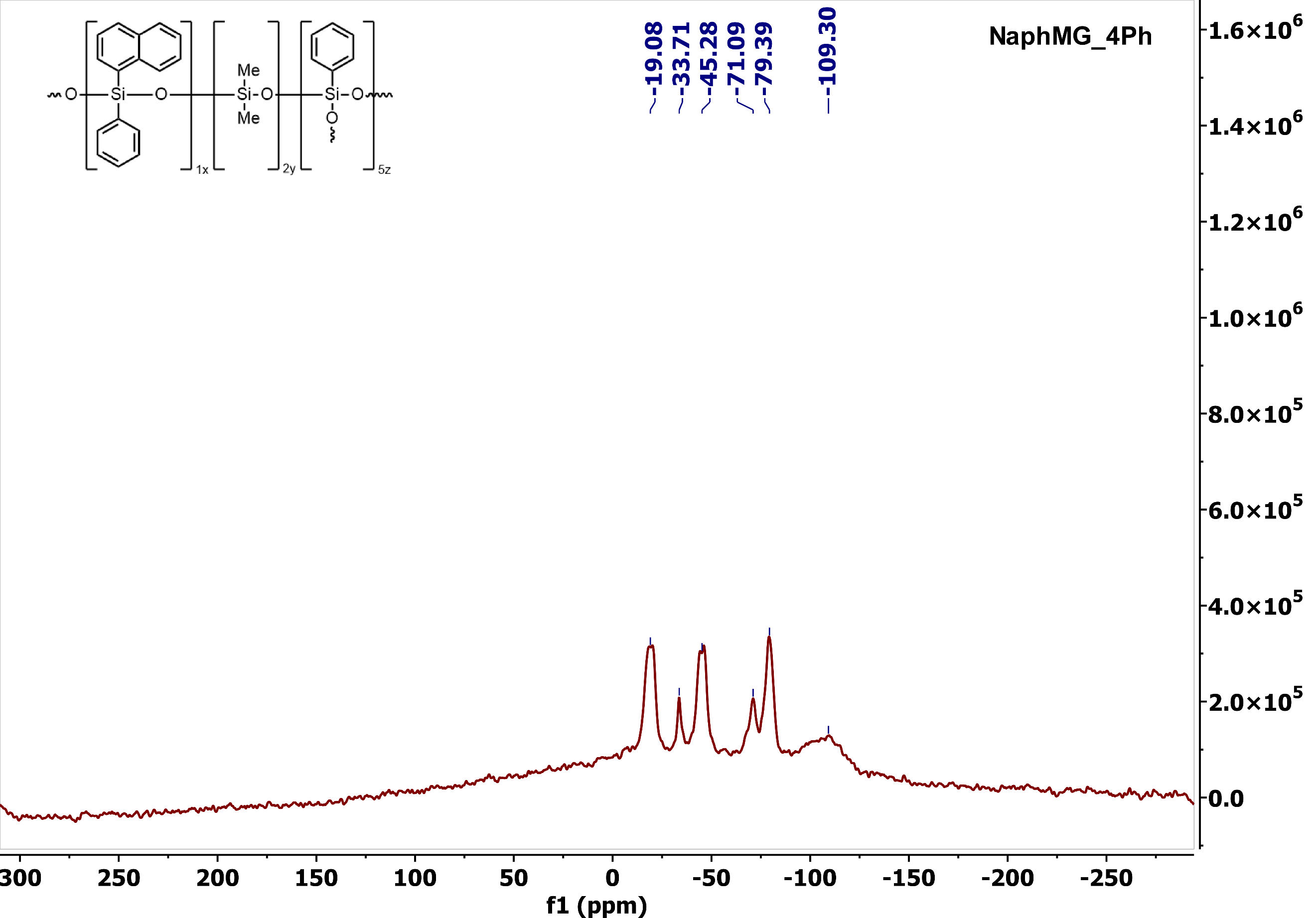


**Figure S14:** ^29^Si MAS spectrum of NaphMG_4Ph after consolidation.


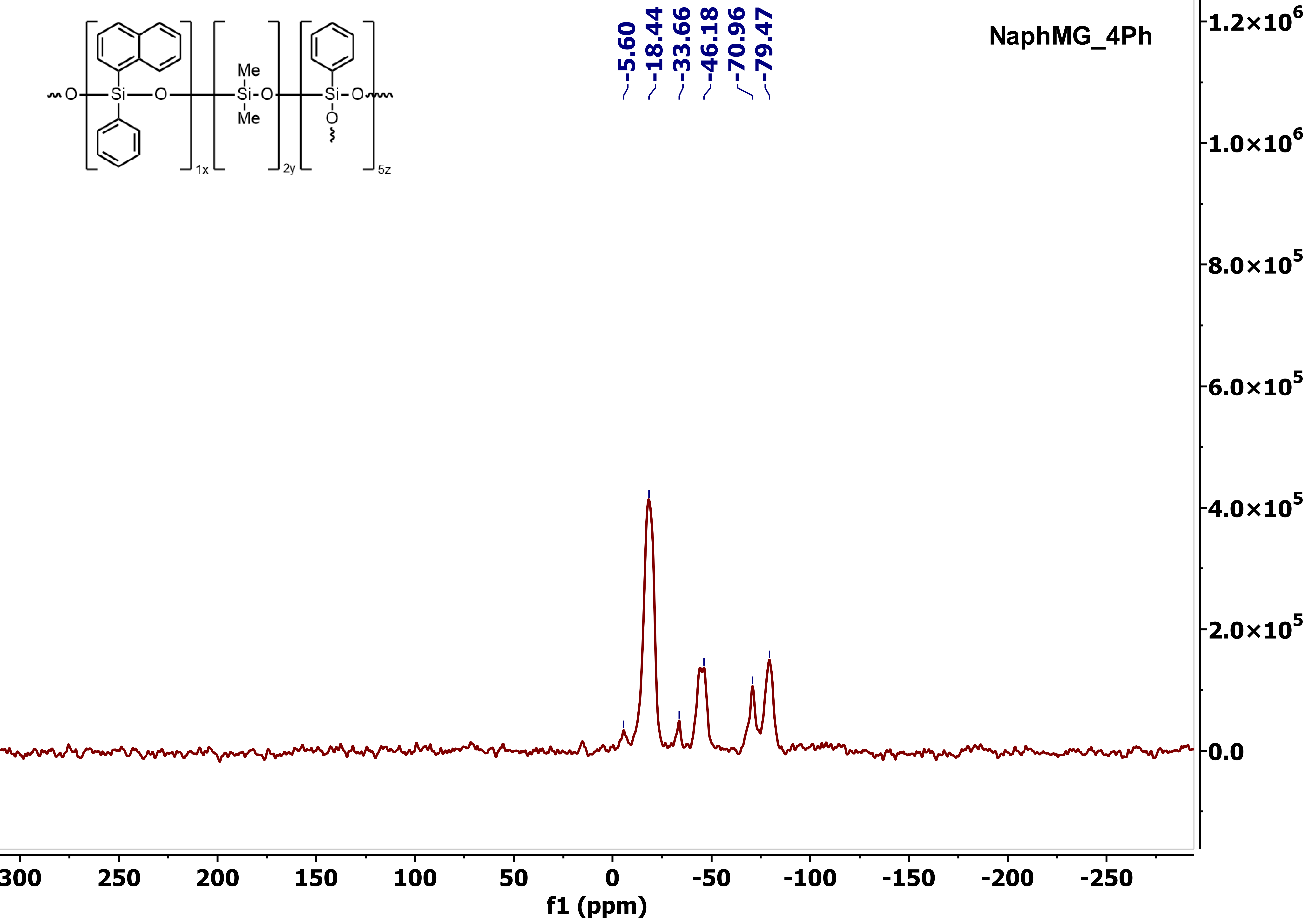


**Figure S15:** ^29^Si CP-MAS spectrum of NaphMG_4Ph after consolidation.





**Figure S16:** Integrated ^29^Si MAS spectrum of NaphMG_4Ph after consolidation.





**Figure S17:** Integrated ^29^Si CP-MAS spectrum of NaphMG_4Ph after consolidation.


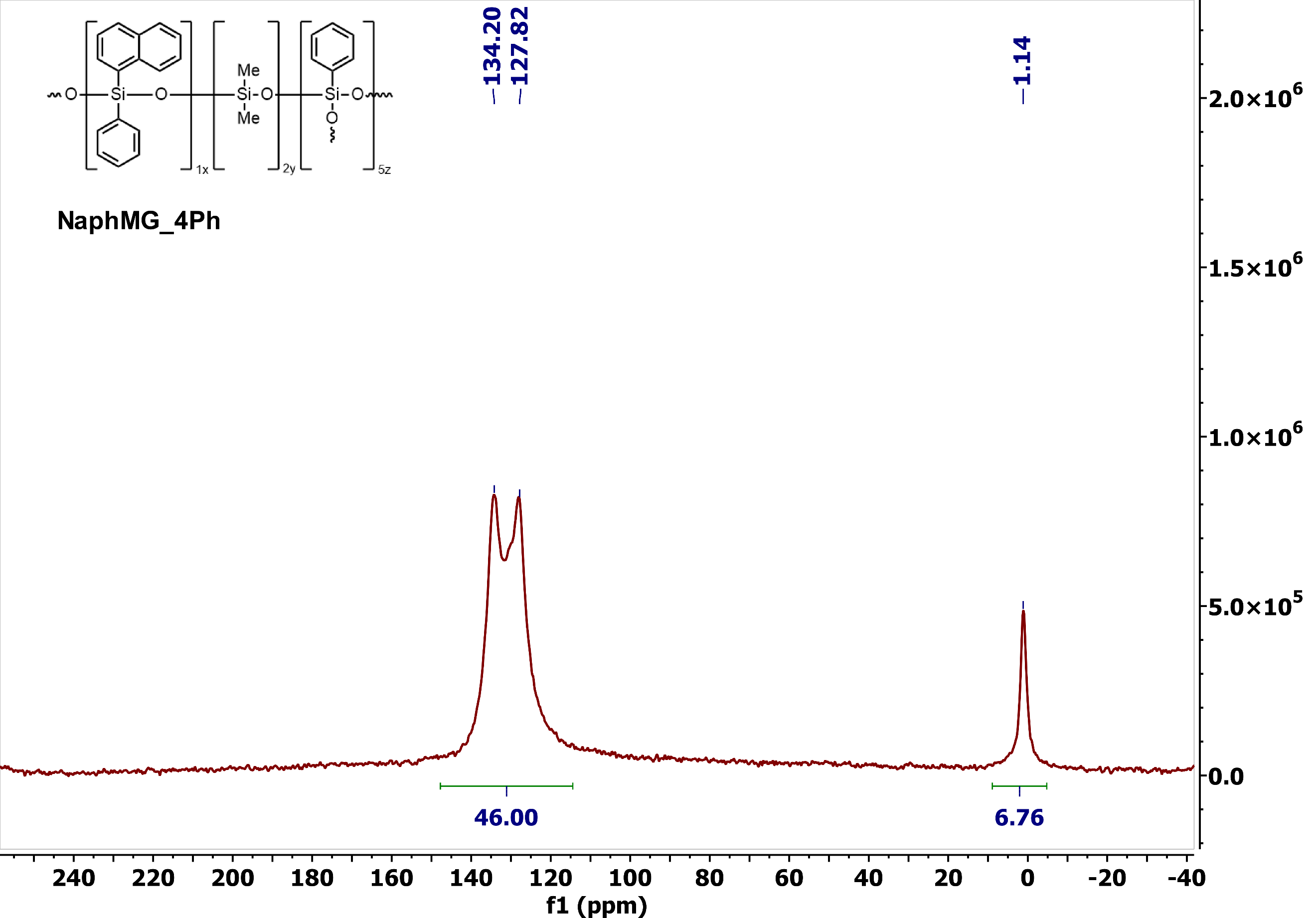


**Figure S18:** ^13^C MAS spectrum of NaphMG_4Ph after consolidation.


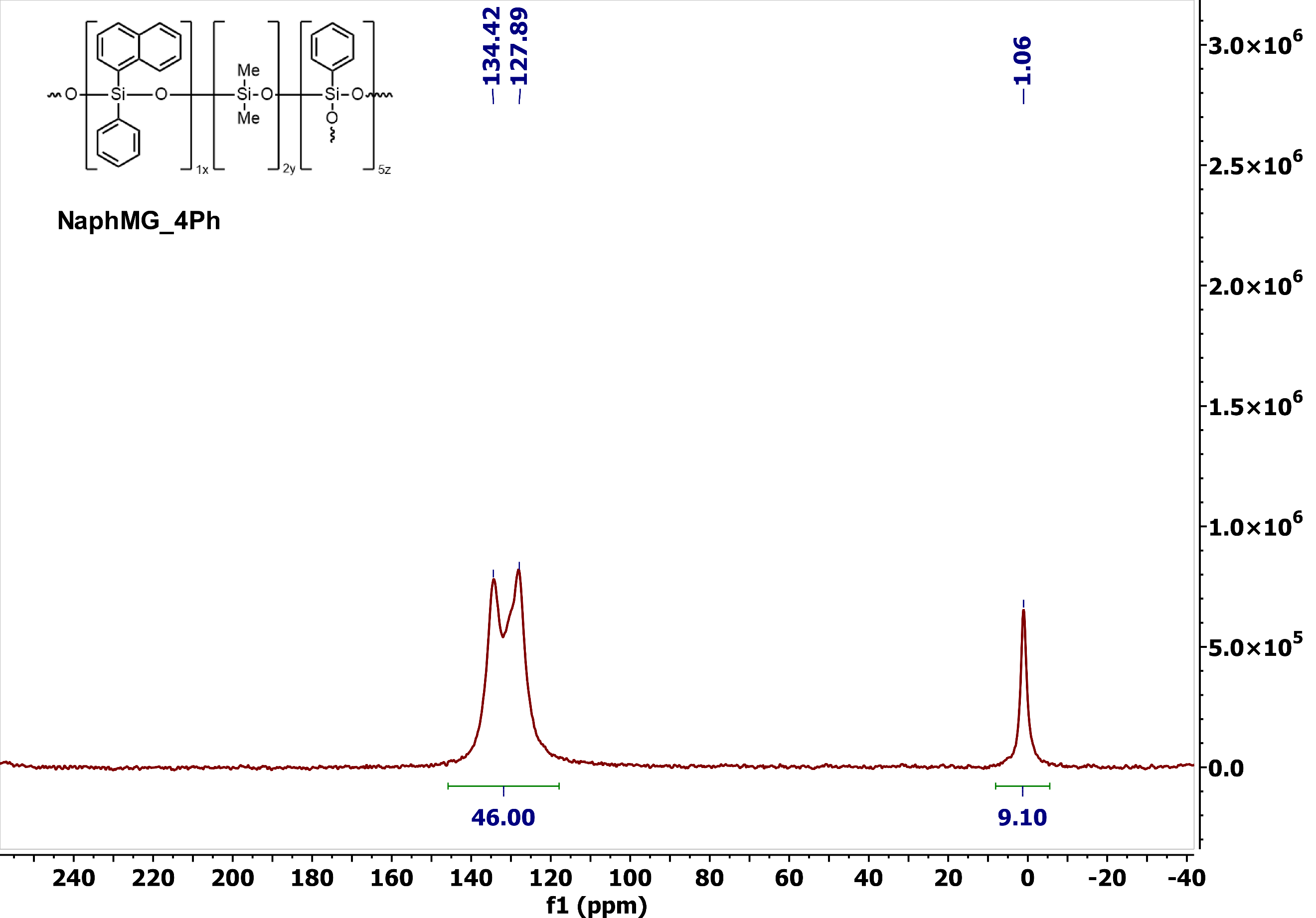


**Figure S19:** ^13^C CP-MAS spectrum of NaphMG_4Ph after consolidation.

**NaphMG_5Ph_d**


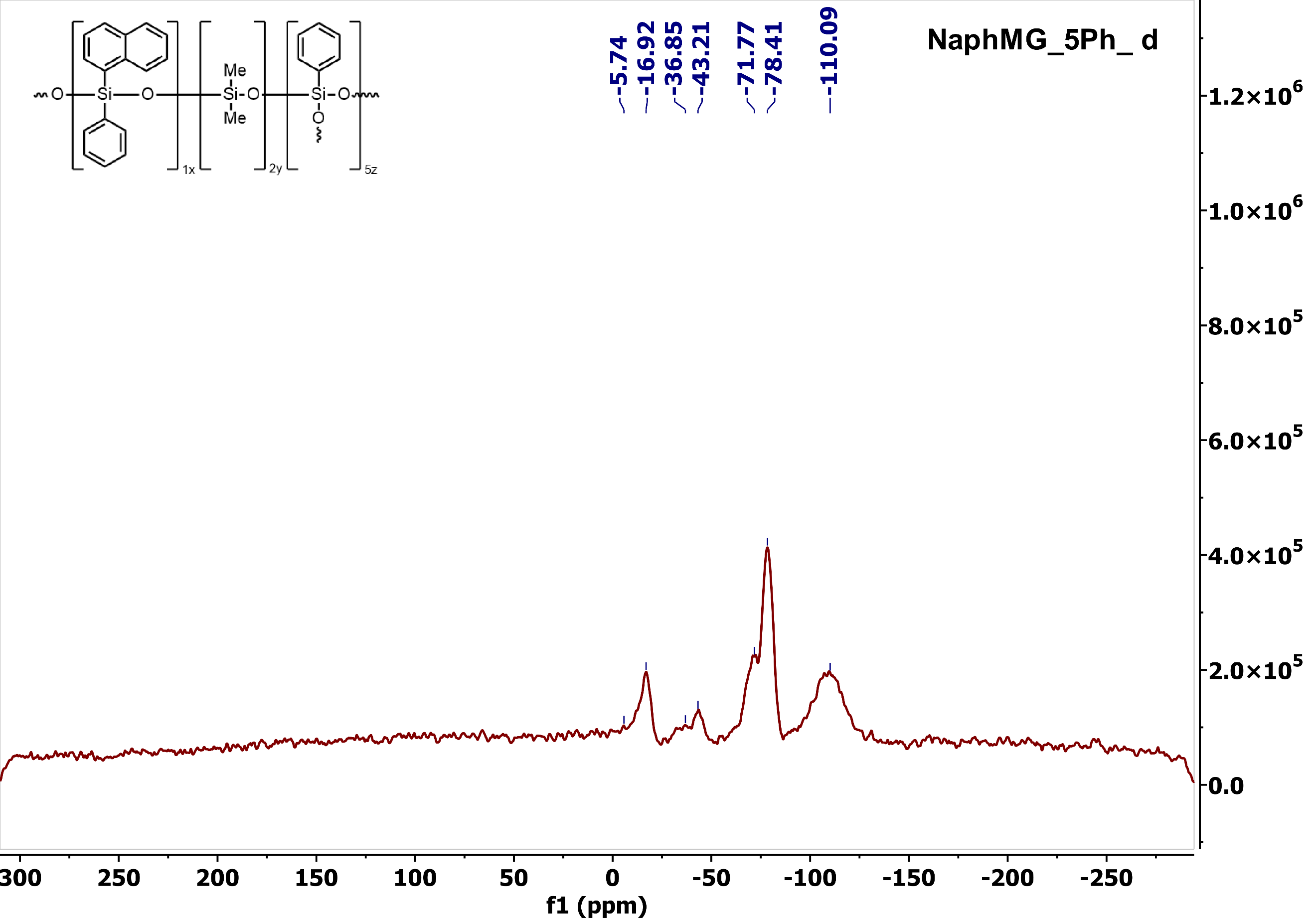


**Figure S20:** ^29^Si MAS spectrum of NaphMG_5Ph_d after consolidation.


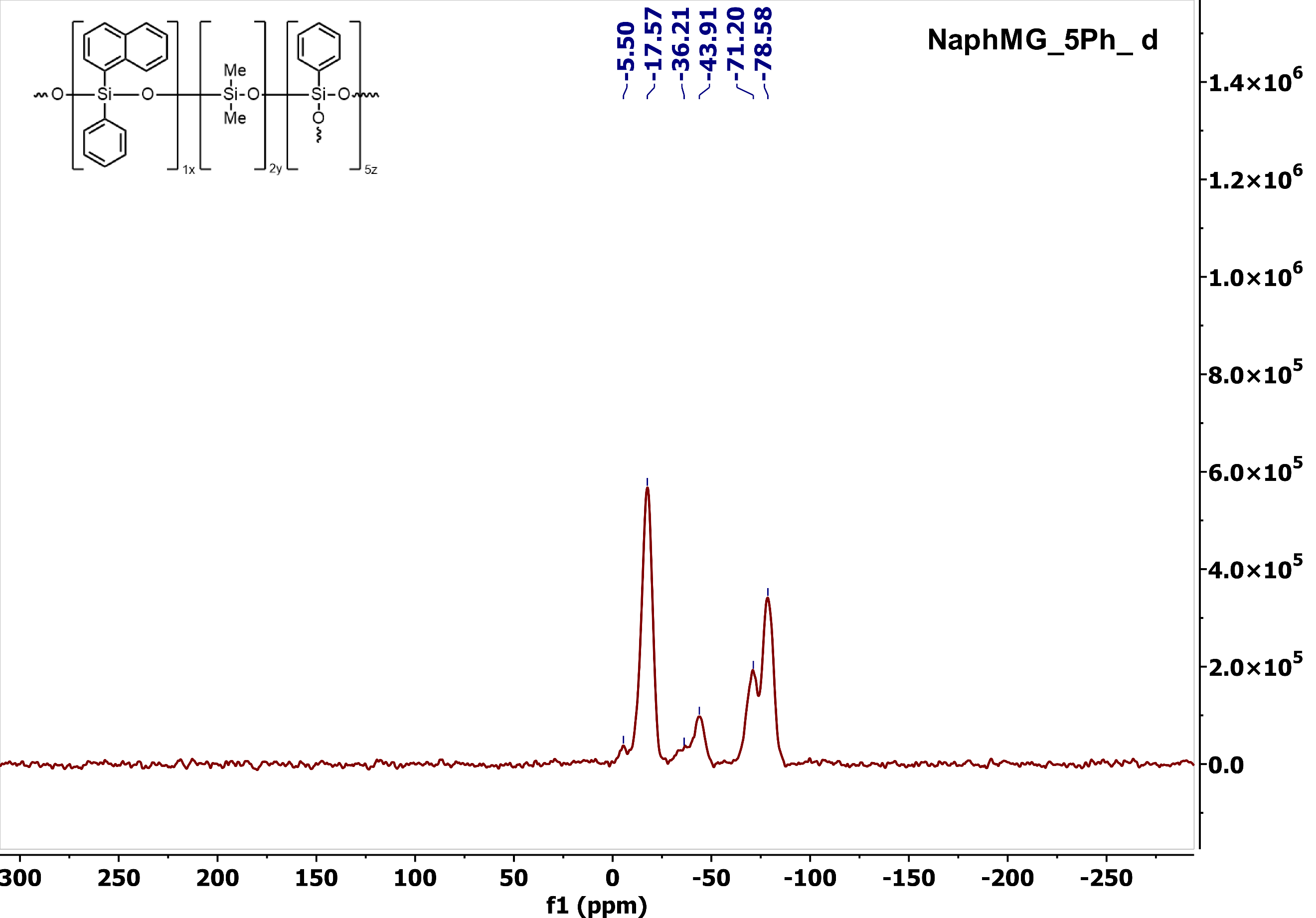


**Figure S21:** ^29^Si CP-MAS spectrum of NaphMG_5Ph_d after consolidation.





**Figure S22:** Integrated ^29^Si MAS spectrum of NaphMG_5Ph_d after consolidation.





**Figure S23:** Integrated ^29^Si CP-MAS spectrum of NaphMG_5Ph_d after consolidation.


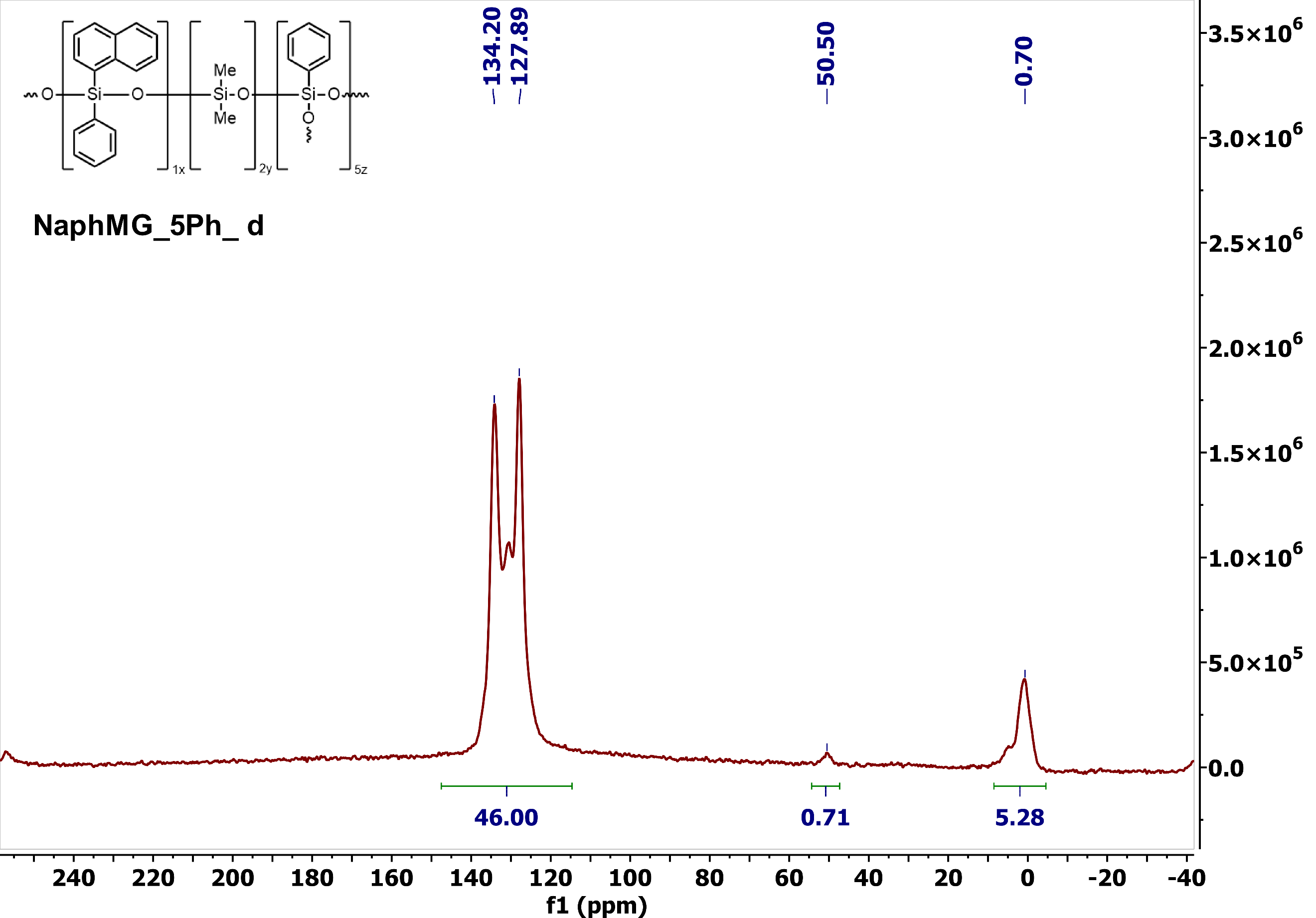


**Figure S24:** ^13^C MAS spectrum of NaphMG_5Ph_d after consolidation.


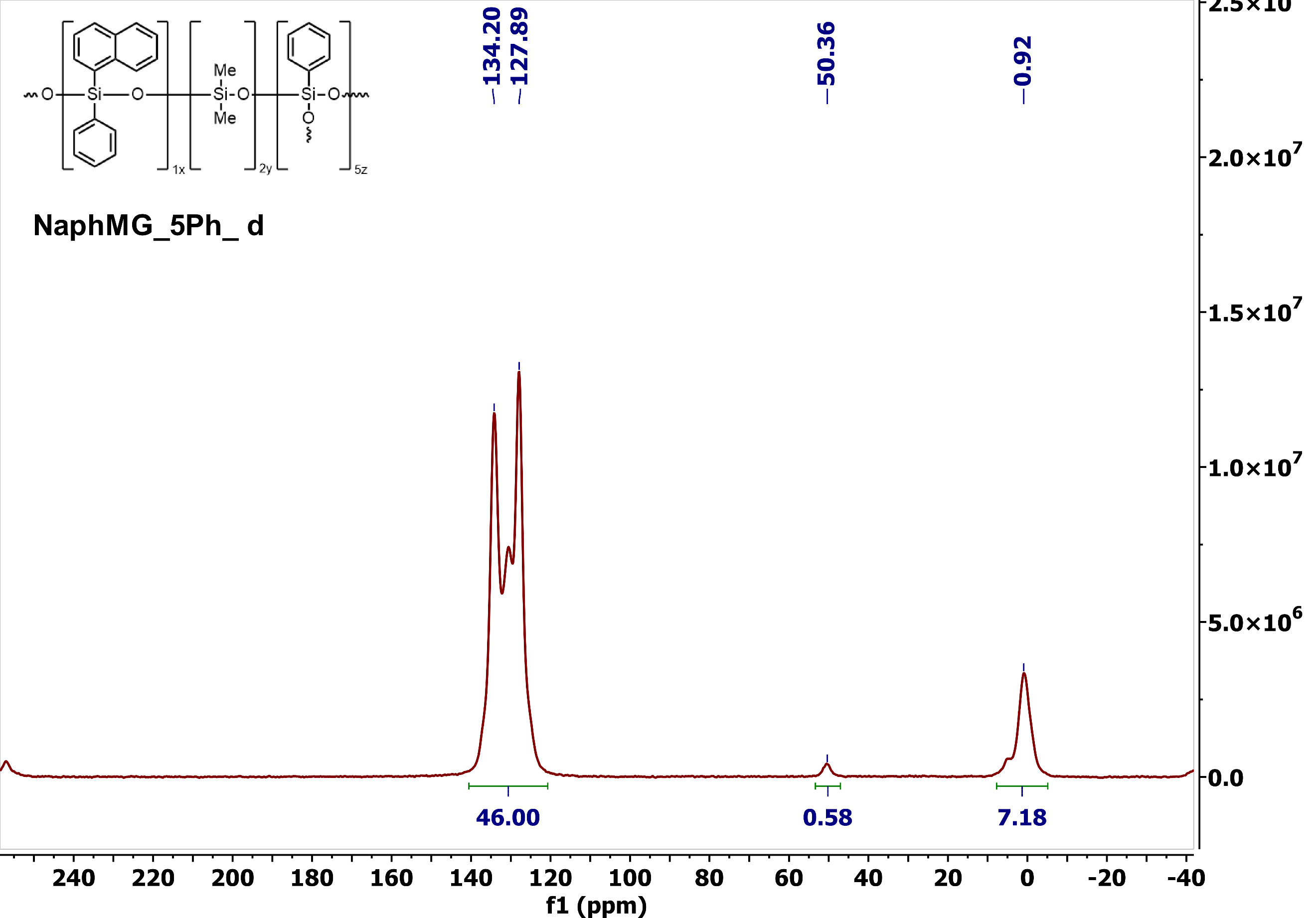


**Figure S25:** ^13^C CP-MAS spectrum of NaphMG_5Ph_d after consolidation.

**Table S2:** Summary of the results of all ^29^Si liquid, ^29^Si CP-MAS and ^29^Si MAS NMR measurements including D‑ and T‑signals as well as DOC.

|  | **NMR** | **D^1‘^ /%** | **D^2‘^ /%** | **D^1^ /%** | **D^2^ /%** | **T^2^ /%** | **T^3^ /%** | **DOC /%** |
| --- | --- | --- | --- | --- | --- | --- | --- | --- |
| **NaphMG_cond.** | Liquid | 0 | 100 | 38.5 | 61.5 | 39.7 | 60.3 | 88.8 |
| **NaphMG_cons.** | Liquid | 0 | 100 | 23.7 | 76.3 | 20.6 | 79.4 | 93.7 |
| **NaphMG_2Me_2_** | Liquid | 0 | 100 | 34.5 | 65.5 | 11.9 | 88.1 | 93.4 |
| **NaphMG_2Ph_2_** | CPMAS | 6.2 | 93.8 | 13.9 | 86.1 | 43.0 | 57.0 | 91.0 |
|  | MAS | 0 | 100 | 18.8 | 81.2 | 17.8 | 82.2 | 94.8 |
| **NaphMG_4Ph** | CPMAS | 6.4 | 93.6 | 14.4 | 85.6 | 34.7 | 65.3 | 92.1 |
|  | MAS | 0 | 100 | 21.2 | 78.8 | 34.6 | 65.4 | 92.0 |
| **NaphMG_5Ph_d** | CPMAS | 5.7 | 94.3 | 32.6 | 67.4 | 41.5 | 58.5 | 88.6 |
|  | MAS | 0 | 100 | 45.1 | 54.9 | 41.6 | 58.4 | 87.6 |

# Fourier Transform Infrared (FTIR) Spectroscopy

**

**

**Figure S26:** FTIR spectra of NaphMG_cond. (black), NaphMG_cons. (red), as well as the consolidated samples NaphMG_4Ph (blue) and NaphMG_5Ph_d (green). Left: full spectrum, right: enlarged area.

**Table S3:** FTIR absorption bands of all samples.^9–12^

| **Absorption band** | **Wavenumber** |
| --- | --- |
| Aryl | 3073 cm^‑1^ (ν_asym_ CH), 3049 cm^‑1^ (ν_sym_ CH), 1591 cm^‑1^ (γ CH_Ar_), 1429 cm^‑1^(ν C-C_Ar_), 845 cm^-1^ (δ CH), 735 cm^-1^ (δ CH),  698 cm^-1^ (δ CH) |
| Methyl | 2962 cm^‑1^ (ν_asym_ CH_3_), 2904 cm^‑1^ (ν_sym_ CH_3_) and 1259 cm^-1^  (δ_s_ CH_3_ (Si-CH_3_)) |
| Si-O-Si | 1131 – 996 cm^-1^, 798 cm^-1^ |
| OH_isolated_ | 3712 – 3575 cm^-1^ |
| OH_H-bonded_ | 3500 – 3120 cm^-1^ |
| Si-OH | 920 – 890 cm^-1^ |

# Powder X-ray Diffraction (PXRD)





**Figure S27:** PXRD spectra of NaphMG_2Me_2_, NaphMG_2Ph_2_, NaphMG_4Ph and NaphMG_5Ph_d. All samples were measured as tablets.





**Figure S28:** PXRD spectra of NaphMG_cond., NaphMG_cons. and NaphMG_Sn. All samples were measured as tablets.

# Ultraviolet-visible (UV-vis) Spectroscopy


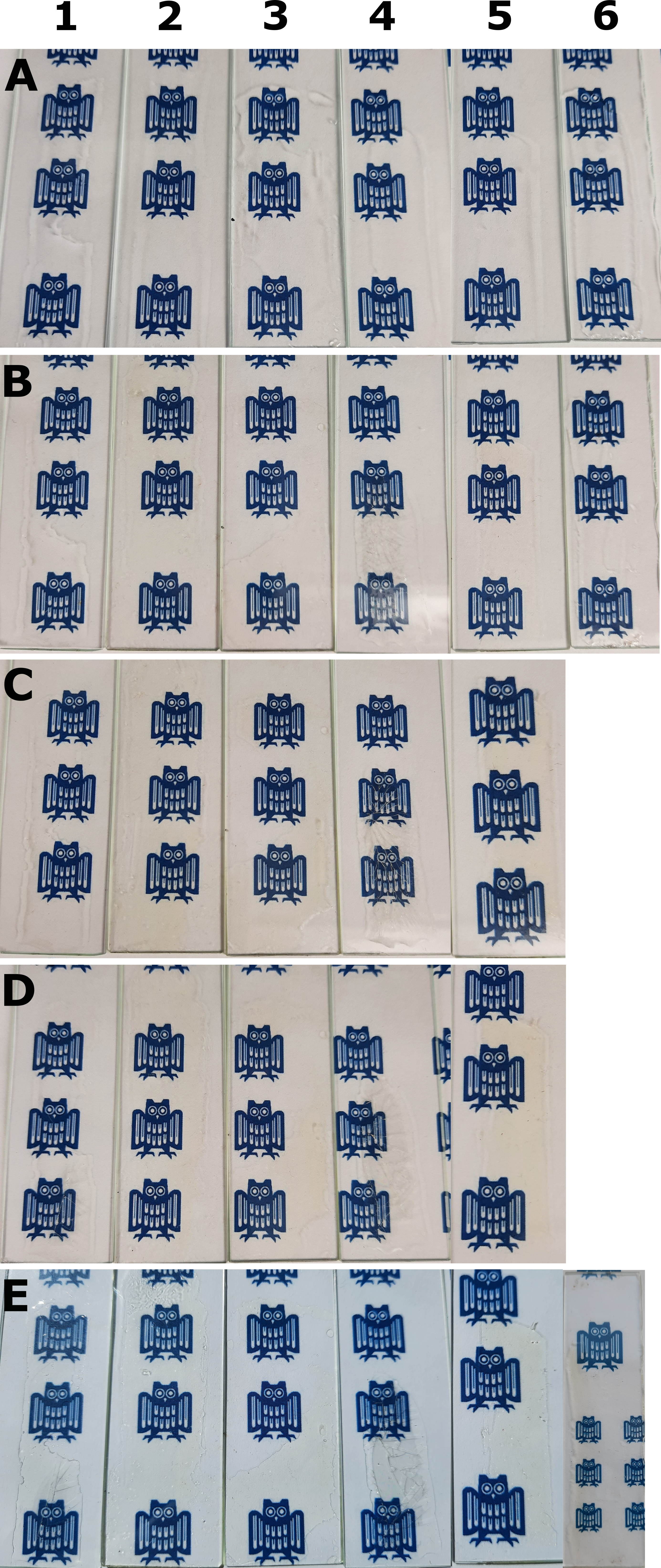


**Figure S29:** Siloxanes doctor bladed onto glass slides. A) before consolidation, B) after consolidation, C) after 3 d at 200 °C D) after 7 d at 200 °C, E) after 7 d at 200 °C, different lighting. 1) NaphMG_2Ph_2_, 2) NaphMG_2Me_2_, 3) NaphMG_4Ph, 4) NaphMG_5Ph_d, 5) NaphMG, 6) NaphMG_Sn.





**Figure S30:** UV-vis measurements of all samples. a) after condensation, b) after 24 h under vacuum at 110 °C, c) after consolidation (72 h, 200 °C), d) after 3 d at 200 °C, e) after 7 d at 200 °C.

# References

(1) Briesenick, M.; Gallei, M.; Kickelbick, G. High-Refractive-Index Polysiloxanes Containing Naphthyl and Phenanthrenyl Groups and Their Thermally Cross-Linked Resins. *Macromolecules* **2022**, *55*, 4675–4691.

(2) Metz, G.; Ziliox, M.; Smith, S. O. Towards Quantitative CP-MAS NMR. *Solid State Nucl. Magn. Reson.* **1996**, *7*, 155–160.

(3) Fyfe, C. A.; Gobbi, G. C.; Kennedy, G. J. Quantitatively Reliable Silicon-29 Magic-Angle Spinning Nuclear Magnetic Resonance Spectra of Surfaces and Surface-Immobilized Species at High Field Using a Conventional High-Resolution Spectrometer. *J. Phys. Chem.* **1985**, *89*, 277–281.

(4) Zhao, X. S.; Lu, G. Q.; Whittaker, A. K.; Millar, G. J.; Zhu, H. Y. Comprehensive Study of Surface Chemistry of MCM-41 Using ^29^Si CP/MAS NMR, FTIR, Pyridine-TPD, and TGA. *J. Phys. Chem. B.* **1997**, *101*, 6525–6531.

(5) Hook, R. J. A ^29^Si NMR Study of the Sol-Gel Polymerisation Rates of Substituted Ethoxysilanes. *J. Non-Cryst. Solids* **1996**, *195*, 1–15.

(6) Cella, J. A.; Cargioli, J. D.; Williams, E. A. ^29^Si NMR of Five- and Six-Coordinate Organosilicon Complexes. *J. Organomet. Chem.* **1980**, *186*, 13–17.

(7) Fyfe, C. A.; Aroca, P. P. Quantitative Kinetic Analysis by High-Resolution ^29^Si NMR Spectroscopy of the Initial Stages in the Sol-Gel Formation of Silica Gel from Tetraethoxysilane. *Chem. Mater.* **1995**, *7*, 1800–1806.

(8) Huang, Y.; Feng, Y.; Sun, X.; Han, Y.; Liu, D.; Tan, X. Preparation of ZrO_2_/Silicone Hybrid Materials for LED Encapsulation via in Situ Sol-Gel Reaction. *Polym. Adv. Technol.* **2019**, *30*, 1818–1824.

(9) Li, Y.-S.; Wang, Y.; Ceesay, S. Vibrational Spectra of Phenyltriethoxysilane, Phenyltrimethoxysilane and Their Sol-Gels. *Spectrochim. Acta - A: Mol. Biomol. Spectrosc.* **2009**, *71*, 1819–1824.

(10) Jitianu, A.; Gonzalez, G.; Klein, L. C. Hybrid Sol-Gel Glasses with Glass-Transition Temperatures below Room Temperature. *J. Am. Ceram. Soc.* **2015**, *98*, 3673–3679.

(11) Sato, Y.; Hayami, R.; Gunji, T. Characterization of NMR , IR , and Raman Spectra for Siloxanes and Silsesquioxanes: A Mini Review. *J. Sol-Gel Sci. Technol.* **2022**, *104*, 36–52.

(12) Hu, N.; Rao, Y.; Sun, S.; Hou, L.; Wu, P.; Fan, S.; Ye, B. Structural Evolution of Silica Gel and Silsesquioxane Upon Thermal Curing. *Appl. Spectrosc.* **2016**, *70*, 1–11.
